# Biomarkers in Rheumatoid Arthritis

**DOI:** 10.7759/cureus.15063

**Published:** 2021-05-16

**Authors:** Samantha C Shapiro

**Affiliations:** 1 Rheumatology, University of Texas at Austin, Dell Medical School, Austin, USA

**Keywords:** rheumatoid arthritis, biomarker, acpa, ccp, rf, esr, crp

## Abstract

The utilization and identification of biomarkers in rheumatoid arthritis (RA) to facilitate timely diagnosis and the optimal management of the disease is an area of active investigation. This review focuses on biomarkers available for routine clinical use, details potential investigational biomarkers, and raises outstanding clinical questions.

## Introduction and background

Rheumatoid arthritis (RA) is a chronic and common systemic inflammatory disease that results in joint deformity and functional disability when not properly managed. The early diagnosis and treatment of RA are imperative for optimal disease control, greater chances of remission, and prevention of permanent clinical and radiographic damage. RA remains a clinical diagnosis although the use and discovery of biomarkers to assist with these goals remain a focus of ongoing research. The 2010 RA Classification Criteria developed by the American College of Rheumatology and European League Against Rheumatism (ACR/EULAR) define a scoring system that includes elements of history, physical exam, and biomarkers that identify patients with RA for the purpose of clinical trial standardization. The criteria have a sensitivity of 84% and specificity of 60% for classification as RA [1-2]. In clinical practice, rheumatologists frequently use these criteria to defend the diagnosis of RA. This review focuses on the four biomarkers included in these criteria that are available for routine clinical use: rheumatoid factor, autoantibodies against citrullinated proteins, erythrocyte sedimentation rate, and C-reactive protein; the multi-biomarker disease activity test is also discussed. A short discussion of investigational biomarkers and outstanding clinical questions follows.

## Review

The current state of the utilization of biomarkers in the diagnosis, prognosis, and management of RA

Rheumatoid Factor (RF)

RFs are autoantibodies directed against the Fc portion of immunoglobulin (Ig) G. In clinical practice, IgM RF is most commonly measured although IgA and IgG RF also exist. RF are found in up to 80% of RA patients but can occur in a myriad of other inflammatory conditions that trigger chronic antigenic stimulation, limiting its specificity. These include but are not limited to other rheumatologic conditions (eg. systemic lupus erythematosus, Sjogren’s syndrome), infectious diseases (eg. hepatitis C virus, subacute bacterial endocarditis, Epstein-Barr virus), malignancy (eg. B-cell neoplasms), and healthy individuals [3]. Smoking has also been associated with an increased prevalence of RF [4]. Approximately 30% to 45% of patients with early RA do not have RF, though some may develop RF later in the course of disease [5].

As with any diagnostic test, the positive predictive value of RF increases when utilized in patients with a high pre-test probability of disease (eg. those with inflammatory arthritis). Testing patients with nonspecific arthralgia, myalgia, or osteoarthritis is not recommended [6]. RF positivity increases the risk of developing RA, with higher titers conferring higher risk [7-8]. RF titers may fall with effective RA therapy but a fluctuation in RF titer does not correlate reliably with disease activity [9]. Serial monitoring of RF levels is not recommended [10-11]. In regards to the selection of RA therapy, RF positivity may increase the chance of response to B-cell depleting monoclonal antibodies (eg. rituximab) [12].

Autoantibodies Against Citrullinated Proteins (ACPA)

Autoantibodies to citrullinated protein epitopes have been a focus of biomarker research in RA for many years. Citrullination is a post-translational modification of proteins that can generate new epitopes to which the immune system is not tolerant, leading to the generation of new autoantibodies [13]. Amongst ACPAs, the assay for anti-cyclic citrullinated peptide (anti-CCP2) is widely clinically available and has excellent diagnostic and prognostic value.

Both RF and anti-CCP2 have similar sensitivities for the diagnosis of RA but anti-CCP2 is more specific [14]. Anti-CCP2 is positive in 20%-30% of RA patients who are negative for RF [15]. A systemic review and meta-analysis that included 37 studies of anti-CCP2 positive patients and 50 studies of RF positive patients showed the pooled sensitivities of RF and anti-CCP2 to be 69% and 67%, and 85% and 95%, respectively [16]. This being said, anti-CCP2 positivity may be found in other rheumatologic diseases (eg. myositis, Sjogren’s Syndrome), especially in the setting of erosive inflammatory arthritis [17]. Anti-CCP2 positivity may also occur with active pulmonary tuberculosis albeit with minimal rheumatologic symptoms [18]. High titer RF and anti-CCP2 antibodies are both associated with an increased risk of erosive joint damage; anti-CCP2 antibodies may confer a higher risk than RF [19-21]. High titer anti-CCP2 is associated with better clinical response to certain biologics (rituximab, abatacept) and thus may aid clinicians in personalizing therapy for the greatest chance of response [22-23].

Erythrocyte Sedimentation Rate (ESR) and C-reactive Protein (CRP)

The ESR, the rate at which erythrocytes fall through plasma when suspended in a vertical tube, is an indirect measure of the levels of acute-phase reactants (mainly fibrinogen). ESR levels are influenced by several factors, such as the size, shape, and number of red blood cells, as well as other plasma constituents like immunoglobulins. Elevated ESR levels may be caused by systemic or local inflammatory processes, infection, malignancy, tissue injury, end-stage renal disease, nephrotic syndrome, and obesity. ESR values increase with age and are slightly higher in women than men. Furthermore, many factors may contribute to spuriously low ESR values, like abnormal erythrocyte shape, extreme leukocytosis, heart failure, and cachexia [24]. Not surprisingly, the ESR is not a specific marker of inflammation.

CRP is an acute-phase reactant in the pentraxin protein family, which comprises pattern recognition molecules involved in the innate immune response [25]. CRP occurs in both acute and chronic inflammatory states, infectious and noninfectious. Low-grade CRP elevation is associated with various metabolic stressors, including but not limited to atherosclerosis, obesity, type 2 diabetes, sedentary lifestyles, unhealthy diet, and even being unmarried [26-27]. CRP levels vary with age, sex, and race, though less so than ESR levels [28]. Furthermore, there is no standardized reference range or unit of measure for CRP values; these vary between laboratories [29]. In the RA synovium, there is an overabundance of pro-inflammatory cytokines that stimulate the production of CRP by the liver, thus making it an attractive candidate as a disease activity biomarker [30]. However, CRP measurement in RA is not foolproof. For example, elevated CRP levels have been independently associated with truncal adiposity in women with RA, regardless of articular involvement or the use of biologic agents [31].

Although ESR and CRP measurements are imperfect, both continue to play a role in the diagnosis and management of RA. Elevated ESR and CRP levels are included in the 2010 ACR/EULAR Classification Criteria for RA [2]. CRP values of less than or equal to 1 mg/dL are included in the 2011 ACR/EULAR definition of RA remission used in clinical trials [32]. The ACR has endorsed six RA disease activity measures for use in clinical practice, two of which include ESR or CRP measurement: the Disease Activity Score 28-ESR or CRP (DAS28-ESR or DAS28-CRP) and the Simplified Disease Activity Index (SDAI) [33]. The 2015 ACR Guideline for the Treatment of RA, widely used in clinical practice, encourages the use of these disease activity measures, though does not specify a preference for measures that include laboratory values over those that do not. The guidelines also do not specifically recommend routine monitoring of ESR and CRP in all RA patients [34]. An update to these treatment guidelines is currently in progress, anticipated fall 2021.

Multiple studies have shown a correlation between ESR and CRP elevation, and radiographic and functional outcomes in patients with RA [30,35]. Elevated ESR is thought to be a better predictor of these outcomes in early RA, whereas CRP may be superior in later stages of the disease given less susceptibility to other factors like immunoglobulin levels and anemia [30]. This being said, ESR and CRP are normal in about 40% of patients with RA [36-37]. Furthermore, even in those patients with baseline elevation, values may remain stable despite clinical improvement with treatment [38]. Interestingly, ESR and CRP values may also be discrepant [24]. A large observational study that included over 9,000 patients from a practice-based registry noted discordant ESR and CRP values in 26% of patients, despite active RA as measured by joint counts and global assessments [39]. When results are discordant, they may no longer predict the progression of radiographic joint damage [40]. Lastly, biologic therapies like tocilizumab, a humanized monoclonal antibody against the interleukin-6 receptor, will normalize CRP values, eliminating utility as a trackable disease activity biomarker.

Multi-Biomarker Disease Activity (MBDA) Test

The MBDA test is a commercially available assay that measures 12 serum protein biomarkers and applies an algorithm to summarize the information into a single score that indicates the level of “RA inflammation.” The following biomarkers are tested: vascular cell adhesion molecule-1 (VCAM-1), epidermal growth factor (EGF), vascular endothelial growth factor A (VEGF-A), interleukin 6 (IL-6), tumor necrosis factor receptor type 1 (TNF-R1), matrix metalloproteinase-1 (MMP-1), matrix metalloproteinase-3 (MMP-3), human cartilage glycoprotein 39 (YKL-40), leptin, resistin, serum amyloid (SAA), and CRP [41]. A 2019 systematic review and meta-analysis identified eight studies that reported correlations between the MBDA and RA disease activity measures currently used in clinical trials and clinical practice. There was a modest correlation between MBDA, DAS28-CRP, and DAS28-ESR, with weaker correlations observed with SDAI, Clinical Disease Activity Index (CDAI), and Routine Assessment of Patient Index Data (RAPID3) [42]. However, subsequent post hoc analysis of data from the AMPLE trial (abatacept versus adalimumab for RA) showed disagreement between the MBDA test score and these measures [43-44]. One trial showed that the MBDA test may be useful in deciding whether or not to continue biologic therapy in the setting of clinical remission [45], and post hoc analyses of data have shown that a high baseline MBDA score is a strong independent predictor of radiographic progression at one year [46-49]. Further study is needed in both regards. At this time, the use of this test is not included in the 2015 ACR Guideline for the Treatment of RA [34]. The cost-effectiveness and role of this test in routine clinical practice remain controversial.

Table 1 summarizes the biomarkers discussed thus far.

**Table 1:** RA biomarkers used in routine clinical practice ACR/EULAR, American College of Rheumatology/European League Against Rheumatism; RF, rheumatoid factor; anti-CCP2, anti-cyclic citrullinated peptide2; ESR, erythrocyte sedimentation rate; CRP, C-reactive protein; MBDA, multi-biomarker disease activity test; DAS28, Disease Activity Score 28; SDAI, Simplified Disease Activity Index

	Diagnostic Biomarkers (2010 ACR/EULAR Classification Criteria for RA)	Disease Activity Monitoring Biomarkers
RF	x	-
anti-CCP2	x	-
ESR	x	DAS28-ESR
CRP	x	DAS28-CRP, SDAI
MBDA	-	Role unclear

Investigational biomarkers in RA

Investigational Biomarkers for Diagnosis

Several biomarkers are currently under study in hopes of improving the accuracy and timeliness of the diagnosis of RA. Approximately 20% to 25% of patients are classified as seronegative RA (negative RF and anti-CCP2 testing). About half of patients are seronegative early in the disease course but eventually become seropositive [14]. Seronegative RA patients experience a delay in diagnosis and delay in initiation of therapy. Hence, they are less likely to attain remission and more likely to suffer joint damage and disability. This suggests a missed “window of opportunity” for intervention (within the first three to six months of illness) [50]. It is unclear whether these patients are truly seronegative, or whether they simply possess RA antibodies that are yet to be identified.

Anti-mutated citrullinated vimentin (anti-MCV), an antibody in the ACPA family, has a similar specificity for RA as anti-CCP2. However, systematic review and meta-analysis of the literature did not reveal superior diagnostic accuracy to anti-CCP2, ultimately limiting the adaptation of anti-MCV testing in routine clinical practice [51-53]. No study has specifically addressed whether adding anti-MCV testing to RF and anti-CCP2 testing would improve overall diagnostic accuracy for RA.

Serum 14-3-3eta, an intracellular chaperonin protein, has been studied as a diagnostic biomarker in RA, but data to date have not been robust enough to defend its routine clinical use. When tested in addition to RF and anti-CCP2, testing may minimally improve rates of diagnosis (from 72% to 78%) or reclassify individuals previously deemed seronegative [54-57]. Further study is needed in this regard.

Antibodies to carbamylated proteins (anti-CarP) have been found in the serum of RA patients. Similar to the other novel biomarkers discussed, studies have not shown increased sensitivity or specificity when compared to the RF and anti-CCP2 testing currently used in clinical practice [58].

Investigational Biomarkers for Disease Activity Monitoring

Given the limitations of ESR and CRP testing as described above, the search for a clinically useful biomarker for disease activity monitoring persists. A biomarker that accurately identifies subclinical disease activity could help guide management decisions and lead to better patient outcomes.

Multiple types of biomarkers are being investigated for the purpose of RA disease activity monitoring: serum acute phase reactants, genetic factors, and tissue-specific markers from cartilage, bone, and synovium. IL-6, a prominent acute phase reactant in RA, remains under investigation but unfortunately has not been found to correlate with the radiographic progression of the disease [59]. Genetic testing may ultimately play a role in the prognosis and selection of therapy given the well-known association of RA with certain human leukocyte antigen (HLA)-DR alleles. Synovium-specific markers of interest include serum hyaluronan, MMP-1, and MMP-3; these have been shown to correlate with radiographic progression [60-61]. Cartilage and bone-specific markers under investigation include serum cartilage oligomeric matrix protein (COMP) and urine C-terminal crosslinked peptides from type I and type II collagen (CTX-I and CTX-II), among others [62-63]. Serum VEGF, a vascular marker, is elevated in RA patients and correlates with radiographic progression [64]. Synovial fluid biomarkers have also been identified, but the clinical utility of these would be limited given the requirement for arthrocentesis to perform testing. Ultimately, the combined use of multiple biomarkers may prove to be a more effective measure of disease activity. Future studies may follow in this regard.

Table 2 summarizes the biomarkers discussed thus far.

**Table 2:** Investigational biomarkers in RA for diagnosis and disease activity monitoring Anti-MCV, anti-mutated citrullinated vimentin; anti-CarP, anti-carbamylated proteins; IL-6, interleukin-6; VEGF, vascular endothelial growth factor; COMP, cartilage oligomeric matrix protein; CTX-I and CTX-II, C-terminal crosslinked peptides from type I and type II collagen; MMP-1, matrix metalloproteinase-1; MMP-3, matrix metalloproteinase-3

Potential Diagnostic Biomarkers	Potential Disease Activity Monitoring Biomarkers
anti-MCV	IL-6
serum-14-3-3eta	serum VEGF
anti-CarP	serum COMP
	urine CTX-I and CTX-II
	serum hyaluronan
	serum MMP-1 and MMP-3
	synovial fluid biomarkers

## Conclusions

To conclude, the biomarkers currently available for the diagnosis, prognosis, and management of RA have several limitations. RF lacks specificity, as any condition that triggers chronic antigenic stimulation may result in positive RF testing. Anti-CCP2 is more specific, but both tests fail to identify 20%-25% of patients with seronegative RA. Disease activity monitoring remains clinical due to the lack of adequate biomarkers for this purpose. ESR and CRP are nonspecific acute phase reactants that may be elevated for a myriad of reasons, and the role of the commercially available MBDA test remains unclear. Novel RA biomarkers of many types (eg. serum, tissue-specific, genetic factors) are under active investigation for both diagnosis and disease activity monitoring. The rheumatology community eagerly awaits data in this regard.

Many outstanding clinical questions remain that better biomarker identification could help answer. Does seronegative RA truly exist, or have we simply not yet identified the antibodies this subset of patients makes? Can we identify a biomarker that permits earlier RA diagnosis, widening the golden three- to six-month “window of opportunity” to therapeutically intervene? Can a universal biomarker be found that accurately identifies ongoing subclinical disease activity, permitting better titration of RA therapies? Can we identify biomarkers that allow for the personalized selection of RA therapies, permitting more rapid and effective disease control? Future work should pursue answers to these questions. Better biomarkers could lead to earlier diagnosis, treatment, and outcomes. Once a better biomarker is identified, the cost and feasibility of testing will need to be considered in order to ensure clinical utility on a worldwide scale. 
